# A Pre-Vaccination Baseline of SARS-CoV-2 Genetic Surveillance and Diversity in the United States

**DOI:** 10.3390/v14010104

**Published:** 2022-01-07

**Authors:** Adam A. Capoferri, Wei Shao, Jon Spindler, John M. Coffin, Jason W. Rausch, Mary F. Kearney

**Affiliations:** 1 HIV Dynamics and Replication Program, Center for Cancer Research, NCI-Frederick, Frederick, MD 21702, USA; jspindler@mail.nih.gov (J.S.); rauschj@mail.nih.gov (J.W.R.); kearneym@mail.nih.gov (M.F.K.); 2 Department of Microbiology and Immunology, Georgetown University, Washington, DC 20007, USA; 3 Advanced Biomedical Computing Science, Frederick National Laboratory for Cancer Research, Frederick, MD 21702, USA; shaow@mail.nih.gov; 4 Department of Molecular Biology and Microbiology, Tufts University, Boston, MA 02129, USA; john.coffin@tufts.edu

**Keywords:** SARS-CoV-2, COVID-19, SARS-CoV-2 evolution, SARS-CoV-2 in United States, variants of concern, viral evolution

## Abstract

COVID-19 vaccines were first administered on 15 December 2020, marking an important transition point for the spread of SARS-CoV-2 in the United States (U.S.). Prior to this point in time, the virus spread to an almost completely immunologically naïve population, whereas subsequently, vaccine-induced immune pressure and prior infections might be expected to influence viral evolution. Accordingly, we conducted a study to characterize the spread of SARS-CoV-2 in the U.S. pre-vaccination, investigate the depth and uniformity of genetic surveillance during this period, and measure and otherwise characterize changing viral genetic diversity, including by comparison with more recently emergent variants of concern (VOCs). In 2020, SARS-CoV-2 spread across the U.S. in three phases distinguishable by peaks in the numbers of infections and shifting geographical distributions. Virus was genetically sampled during this period at an overall rate of ~1.2%, though there was a substantial mismatch between case rates and genetic sampling nationwide. Viral genetic diversity tripled over this period but remained low in comparison to other widespread RNA virus pathogens, and although 54 amino acid changes were detected at frequencies exceeding 5%, linkage among them was not observed. Based on our collective observations, our analysis supports a targeted strategy for worldwide genetic surveillance as perhaps the most sensitive and efficient means of detecting new VOCs.

## 1. Introduction

The first reported case of COVID-19 in the U.S. was on January 20th, 2020 [1]. Since that time, through to the beginning of January 2022, there have been more than 57 million U.S. cases (19.3% of global) and 828,000 deaths (15.2% of global) [2]. By late 2020, several vaccines had been developed, tested, and approved, and since Dec 15, 2020, have been widely administered in the U.S. and the world. The end of 2020 was also marked by the emergence of SARS-CoV-2 variants with greater transmissibility, virulence, and partial resistance to current preventives and treatments [3,4]. Though they share some common genetic features, such ‘variants of concern’ (VOCs) appear to have emerged independently in different regions throughout the world [5,6]. Among these, the Delta VOC, which began to spread rapidly in the U.S. in early 2021, became the dominant form in the U.S until the emergence and spread of the Omicron VOC. In this work, we provide a detailed characterization of the early spread of SARS-CoV-2 in three phases across the different geographic regions of the U.S. and assess the varying levels of genetic surveillance and genetic diversity in each region and phase. Assessing the spread and genetics of SARS-CoV-2 in the U.S. in 2020, largely in the absence of immune selection pressure from prior infection or vaccine administration, will allow us and others to contrast these patterns with the surveillance and genetics of the virus in the future, with emphasis on improving the early detection of new variants.

## 2. Materials and Methods

### 2.1. Sources for COVID-19 Cases and Deaths in the U.S. in 2020

To characterize the early spread of SARS-CoV-2 across different regions in the U.S., we extracted data for U.S. COVID-19 cases and deaths between 1 January 2020 and 15 December 2020 from https://COVIDtracking.com/data (accessed 17 December 2020) [7] or https://COVID.cdc.gov (accessed 5 January 2022) [2]. U.S. regions were assigned based on the four Census Regions of the United States (U.S. Census Bureau) [8]. Viral spread in the U.S. was divided into three phases based on COVID-19 case peaks where the derivative of the trough was approximately zero: Phase 1 of winter–spring (1 January 2020–31 May 2020), Phase 2 of summer (1 June 2020–31 August 2020), and Phase 3 of fall (1 September 2020–15 December 2020). The estimated 2019 population for each U.S. state was accessed by the U.S. Census Bureau (Supplementary Materials Table S1) to normalize the incidence of COVID-19 cases and deaths in the sub-regional areas. GraphPad Prism V.8.4.3 was used to visualize the data.

### 2.2. Sources and Numbers of SARS-CoV-2 Sequences Used in Surveillance and Diversity Analyses

A total of 36,299 full-length (29,782 bp), high-coverage SARS-CoV-2 genomes from humans in the U.S. with infections between 20 January 2020 and 15 December 2020 were obtained from gisaid.org (accessed on 18 December 2020) [9,10] for surveillance and sequence analysis. The numbers of sequences obtained were: Phase 1 (22,434), Phase 2 (11,893), and Phase 3 (2072). All sequences used in the analyses can be found in the Supplemental Dataset. To minimize miscounting of mutations in our sequence alignments, we replaced rare instances of the standard ambiguous base designation ‘N’, which would be considered a match with any nucleotide (i.e., A, T, C, G), and thus artificially reduce the apparent mutation rate, with gaps, so that no comparison with the reference at these positions would be made. SARS-CoV-2 Clade O, Clade GV, Cruise-ship, and other U.S. territory sequences were excluded from the analysis due to the limited number of sequences. Sequences from VOCs: Alpha (Pango lineage B.1.1.7, Nextstrain 20I, GISAID clade GR, originally isolated in the U.K.), Beta (B.1.351, 20H, GH, South Africa), Gamma (P.1, 20J, GR, Brazil), and Epsilon (B.1.427+B.1.429, 21C, GH, United States) were accessed from gisaid.org on 1 April 2021; and Delta (B.1.617.2, 21A, GK, India) were accessed from gisaid.org on 27 July 2021 (see also Section 2.5) [9,10,11]. The VOCs (Alpha, Beta, Gamma, and Epsilon) were sampled in the U.S. between 1 November 2020 and 31 March 2021 because of their earlier introduction or emergence in the U.S compared to Delta. All VOC sequences available at the time were accessed resulting in a dataset with 37 Alpha sequences, 38 Beta, 26 Gamma, and 31 Epsilon VOC. An additional 15,733 Delta sequences were added to the final dataset corresponding to the later detection of the Delta VOC. Gap-stripped alignments were generated using the FFT-NS-1 200PAM/k = 2 algorithm of MAFFT v7.450 [12,13]. Additional analyses and data handling of SARS-CoV-2 sequences were conducted using Geneious Prime^®^ 2020.2.4 and several in-house-generated Perl scripts available at https://github.com/Wei-Shao/COV2-Analysis(accessed 17 December 2020).

### 2.3. Analysis of SARS-CoV-2 Genetic Surveillance in the U.S. in 2020

To assess the relationship between the case distribution and genetic surveillance of SARS-CoV-2 in the U.S., genomic sequences from each phase were separated by region and clade for each month in 2020. To estimate the number of COVID-19 cases within each GISAID clade, the number of sequences was multiplied by the total monthly new COVID-19 cases. RStudio v1.3 [14] and GraphPad Prism V.8.4.3 were used to tabulate data and for data visualization.

The level of SARS-CoV-2 genetic surveillance in the U.S. in 2020 was also compared to levels in other developed nations in the same time period (U.K. and in Australia). Sequence data from the U.K. and Australia were obtained under the same selection criteria as for the U.S. The number of cases was accessed on the same days using https://coronavirus.data.gov.uk/ (accessed 17 December 2020) [15] and the National Notifiable Diseases Surveillance System (http://www9.health.gov.au/cda/source/rpt_3.cfm, accessed 17 December 2020) [16]. The level of sequencing was determined from the number of sequences obtained monthly and the number of monthly cases of COVID-19 using an in-house generated bioinformatic script to visualize data (script available at https://github.com/aacapoferri/COV2, accessed 18 December 2020).

### 2.4. Mutation Detection and Measurements of Genetic Diversity and Divergence

Sequences obtained as described above were used to detect the emergence of new SARS-CoV-2 mutations and to assess the clade genetic diversity and divergence across the 3 phases of spread in the U.S. in 2020. All sequences for each clade and phase were included in each analysis with the exception of clade GH, where the number of sequences was too high for measurements of genetic diversity and divergence and, therefore, 2500 sequences were randomly subsampled.

SARS-CoV-2 mutation frequencies were determined for each clade/phase compared to either the majority-rule of Phase 1 consensus sequence, the Wuhan-Hu-1 reference genome (GenBank accession, NC_045512.2), or the VOCs [17]. To exclude clade-defining mutations and amplification/sequencing errors, several steps were taken. First, majority-rule consensus sequences for each clade were generated from all genomes in Phase 1 and used as a reference for detecting new mutations that emerged in Phases 2 and 3. This approach allowed majority clade-associated mutations to be omitted for the detection of new mutations only. Second, a threshold ≥5% frequency was used to eliminate mutations that were rarely detected and, therefore, could be PCR errors, sequencing errors, or real but not determined to be sustained in the population with at least 95% confidence (see Section 3.4) [18]. Our approach to setting a threshold was as follows: first, the 99th percentile for all clades (G/GH/GR/L/S/V) across Phases 1, 2, and 3 for all non-zero mutation frequencies at each nucleotide position was calculated (average of 3.84%); second, to account for error mutations, the upper–outer fence [Q3 + 1.5IQR] of the 99th percentiles was calculated (6.09%); finally, to resolve this range, the median was rounded to the nearest whole percent (5%). Mutation frequencies were plotted and annotated using the “Mutation frequency for SARS.R” script (an example provided on https://github.com/aacapoferri/COV2, accessed 17 December 2020). Mutations that were present in any phase at ≥5% of the population were noted for each G-based clade and in each phase. Heatmaps were generated using GraphPad Prism V.8.4.3 to visualize the persistence and emergence of mutations that were present in at least one phase in greater than ≥5% of the surveyed populations.

Mutation distributions were determined by assessing the number of mutations per sequence for each clade during each phase by Hamming distance. To examine the number of mutations per sequence in the G-based clades during each phase, the distribution of the number of mutations relative to the Wuhan-Hu-1 reference genome was generated using an in-house script available at https://github.com/Wei-Shao/COV2-Analysis (accessed 17 December 2020).

Statistical shifts in population structure (divergence) were determined using a test for panmixia with a statistical cut-off at *p* < 10^−3^ [19]. Population genetic diversity was calculated as average pair-wise distance (APD) in MEGAX for each clade/phase [20]. APD was determined using p-distance and included transitions/transversions with rates among sites as uniform where gaps/missing data were treated as a complete deletion. All sequences were used for each clade/phase for calculating APD, except in clade GH during Phases 1 and 2, where random subsampling of 2500 sequences was performed. When calculating the APD for each clade per month, 50 sequences were randomly subsampled in triplicate. Where there were fewer than 50 sequences available for a given clade/month, all sequence were utilized unless there were fewer than 10, in which case that clade/month was excluded from the analysis.

Identical SARS-CoV-2 genomes were collapsed to determine the number of different variants in the dataset. A simple linear regression was determined for clades G, GH, and GR in GraphPad Prism V.8.4.3 with the linear equation and goodness-of-fit (R^2^) reported. The slope was understood as the rate of change in %APD/month. The length of the SARS-CoV-2 genome is ~30,000 base pair, which when multiplied by the slope, gave an approximate number of nucleotide changes/month for a given G-based clade.

To examine mutations in mapped epitope sites, majority-rule consensus sequences were generated for the G-based clades in each phase and were aligned to the Wuhan-Hu-1 reference genome. For comparison and fine-mapping, previously described T-cell and B-cell epitopes from Spike and Nucleocapsid are listed and referenced in Table S2. Nonsynonymous mutations observed in the G-based clades that differed from the reference in either T-cell or B-cell epitopes were noted. To distinguish between purifying and positive selection, the dN/dS ratio was calculated for each codon position in Spike and Nucleocapsid using an in-house Perl script at https://github.com/Wei-Shao/COV2-Analysis (accessed 17 December 2020).

### 2.5. Analyses of Variants of Concern (VOCs)

The five VOCs used in this study included the Alpha, Beta, Gamma, Delta, and Epsilon sampled within the U.S. The Delta VOC was assigned as GISAID clade GK (defined after 2020). The numbers of mutations per sequence were determined for each VOC and compared to the corresponding values calculated for the GISAID clades during Phase 3. The APD for each VOC dataset was calculated and compared to the APDs of each G-based clade in the U.S. during 2020. Because the SARS-CoV-2 Spike protein is particularly important for vaccine and therapeutic strategies, we searched for and characterized VOC-defining S gene mutations in the U.S. viral population even when their frequencies did not meet our 5% threshold.

### 2.6. Phylogenetic Analysis

We reconstructed a phylogenetic tree using a random subsample of 300 sequences for each clade (G/GH/GR) and sequences from the circulating VOCs, as described in the dataset. Sequencing depth was an important consideration due to the random subsampling and the relatively small sequence dataset for several VOCs. From our analysis in Figure S5, we found that we could detect mutations present at frequencies ≥1% with 95% probability using datasets of 300 sequences. An alignment of 1,084 sequences was generated with MAFFT v7.450 (FFT-NS-1 200PAM/k = 2 algorithm) [12,13]. The final alignment was 28,744 bp in length after ends were trimmed. A maximum-likelihood phylogeny was estimated with RAxML-NG v.1.0.0 [21,22] using the GTR+I+G4 substitution model after optimizing with ModelTest-NG v0.1.7 [23] in raxmlGUI 2.0 [24]. Optimized model parameters were as follows: −lnL = 101,634.84, AIC_C_ = 215,879.42, freq[A, C, G, T] = [0.30, 0.17, 0.18, 0.35], R[A-C, A-G, A-T, C-G, C-T, G-T] = [0.16, 0.73, 0.13, 0.11, 2.47, 1.00], Among-site rate variation by proportion of invariable sites (I) = 0.63, and Variable sites (G) with Gamma distribution shape parameter = 1.01. The outgroup was set to the Wuhan-Hu-1 reference genome. Tree visualization was performed in FigTree v1.4.4 (http://tree.bio.ed.ac.uk/software/figtree/, accessed 17 December 2020).

### 2.7. Viral Genetic Surveillance Resources

Several databases were used to compare and contrast global trends and our U.S.-based specific analysis. These included: PANGO lineages (https://cov-lineages.org/, accessed 17 December 2020) [25], NextStrain (https://nextstrain.org/sars-cov-2/, accessed 17 December 2020) [26], Global Initiative on Sharing Avian Influenza Data (https://www.gisaid.org/, accessed 17 December 2020) [9,10], Outbreak.info (https://outbreak.info/situation-reports, accessed 17 December 2020) [27], Observable (https://observablehq.com/@spond/linkage-disequilibirum-in-sars-cov-2, accessed 17 December 2020), Virological forum (https://virological.org/, accessed 17 December 2020), Los Alamos National Laboratory (https://cov.lanl.gov/content/index, accessed 17 December 2020), and the U.S. Centers for Disease Control and Prevention (https://www.cdc.gov/coronavirus/2019-ncov/cases-updates/variant-surveillance/variant-info.html, accessed 17 December 2020).

## 3. Results and Discussion

### 3.1. Spread of SARS-CoV-2 in the U.S. Pre-Vaccination Did Not Coorelate with Geographic or Levels of Sequence Surveillance

**Figure 1:**
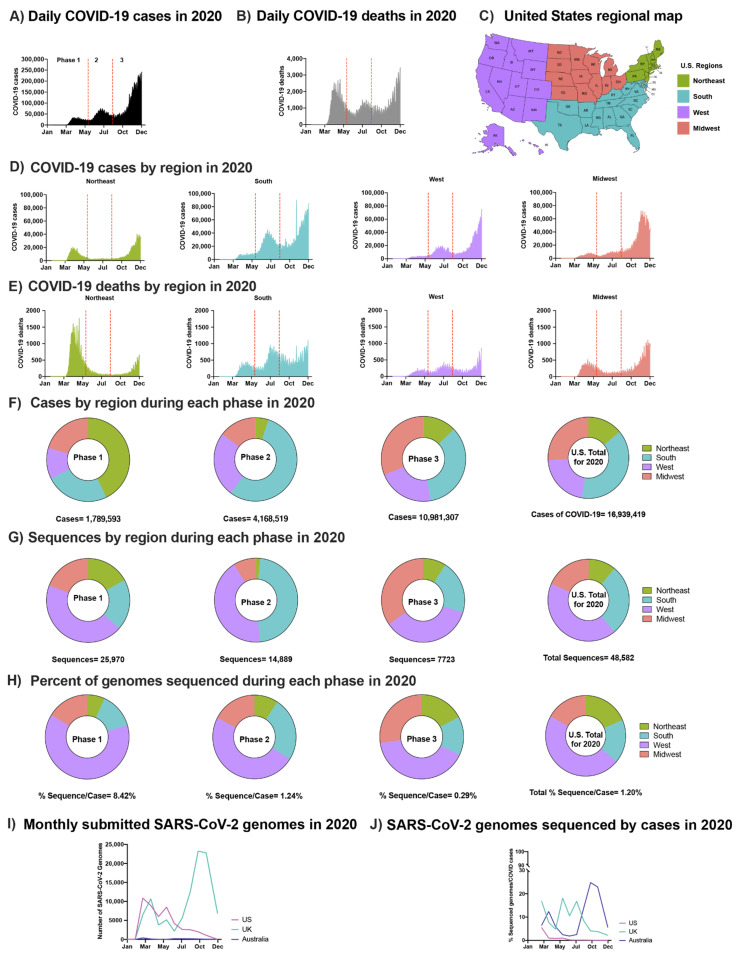
SARS-CoV-2 epidemic in the U.S. in 2020: (**A**) Daily COVID-19 cases in the U.S. in 2020. (**B**) Daily COVID-19 deaths in the U.S. in 2020. (**C**) U.S. regional map colored by region. (**D**) Number of COVID-19 cases in the U.S. in 2020 by region: Northeast, South, West, Midwest, respectively. (**E**) Number of COVID-19 deaths in the U.S. in 2020 by region. (**A**,**B**) and (**D**,**E**) Separation of phases is denoted by vertical dotted red lines. Data were smoothed by a moving 3-day average. (**F**) Proportion of COVID-19 cases by region during each phase and the overall contribution to the U.S. total in 2020. (**G**) Proportion of SARS-CoV-2 sequences accessed (submission as of 15 December 2020) by region during each phase and the overall contribution to the U.S. total in 2020 (**H**) The number of sequences per case were obtained by each region during each phase and the U.S. total in 2020. (**F**–**H**) Highlights Phases 1, 2, and 3, followed with U.S. total of 2020. (**I**) Total number of sequences submitted to GISAID from the U.K., Australia, and the U.S. by 15 December 2020. (**J**) Submitted SARS-CoV-2 genomes normalized to the number of COVID-19 cases from the U.K., Australia, and the U.S. (see Section 2.3).

To characterize the early spread of SARS-CoV-2 in the U.S., we compared the cases and deaths across each geographic region (Figure 1). The spread of SARS-CoV-2 in the U.S. in 2020 occurred in three phases marked by peaks in case numbers, hospitalizations, and deaths (Figure 1A,B). Phase 1, in the winter and spring of 2020, began with the introduction of SARS-CoV-2 from Europe and Asia [28] and was followed by a surge in cases resulting from community spread and mobility [29], especially in the northeast (Figure 1C–E) [30,31,32,33]. Phase 2 began in early June with accelerated community spread primarily in the south and west after mitigation policies were relaxed (Figure 1D,E). The start of Phase 3 in the fall of 2020 was marked by a surge in transmission in the Midwest (Figure 1D,E), followed by a nationwide increase, at or near the end of which time public vaccination was initiated (Figure S1 and Table S1).

The regional distribution of COVID-19 cases varied by phase and was not always correlated with the level of viral sequencing in the different regions (Figure 1G,H). For example, although the south had the greatest overall number of cases (Figure 1F), the majority of SARS-CoV-2 sequences were obtained from samples collected in the west (Figure 1G,H), an observation made by a previous group as well [34]. Multiple factors contributed to this disproportionate sequencing including the failure to initiate a national genetic surveillance plan early in the pandemic, limited funding for sequencing, and limited access to donor samples in some regions of the country. In total, viral sequences were obtained from 1.2% of reported U.S. cases in 2020, a low level compared to other developed nations such as the U.K. (8.1%) and Australia (6.2%) (Figure 1I,J). The aggregate rate of sequencing in the U.S. reflects a decrease from 8.4% in Phase 1 to 0.3% in Phase 3, a difference that can be partly explained by the long intervals between sample collection and sequence deposition in GISAID (median: ~100 days; Figure 1H,I). Since vaccines were first administered in late 2020, the rates of SARS-CoV-2 sequencing in the U.S. have increased significantly. Though this development is certainly encouraging with respect to early detection of emerging variants and increased efforts to better match case and sequencing geographical distributions, more targeted approaches are still needed to detect the evolution of new VOCs.

### 3.2. SARS-CoV-2 Genetic Diversity and Divergence Increased in the U.S. in 2020

**Figure 2:**
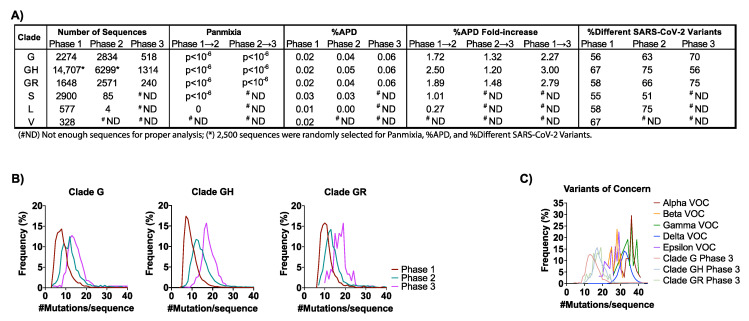
SARS-CoV-2 genetic diversity increased over time: (**A**) The number of sequences obtained from GISAID (https://www.gisaid.org/, accessed 17 December 2020) in the analysis for each clade by phase are reported. Genetic divergence was measured by panmixia probability [40] (significance cutoff, *p* < 10^−3^) for clade/Phase with >11 sequences for Phase 1 → 2 and Phase 2 → 3. Genetic diversity was measured by average pair-wise distance (%APD) and the percent of different SARS-CoV-2 variants. (**B**) The distribution of the frequency for the number of mutations per sequence relative to the Wuhan-Hu-1 isolate was determined for the G-based clades in each Phase. (**C**) Distribution of the frequency for the number of mutations per sequence for the VOCs: Alpha, Beta, Gamma, Delta, and Epsilon. The number of mutations/sequence (mut/seq) at the maximum peak frequency were: Clade G (13 mut/seq), Clade GH (17 mut/seq), Clade GR (19 mut/seq), Alpha VOC (36 mut/seq), Beta (28 mut/seq), Gamma (36 mut/seq), Delta (32 mut/seq), and Epsilon (29 mut/seq). (^#^ ND) Not enough sequences for proper analysis; (*) 2500 sequences were randomly selected for Panmixia, %APD, and %Different SARS-CoV-2 Variants.

To characterize the increasing genetic diversity and divergence of SARS-CoV-2 in the U.S. prior to both the introduction of vaccines and detection of the first VOC, high-coverage full-length genome sequences with GISAID submission dates on or before 15 December 2020 were analyzed. All early GISAID-assigned clades of SARS-CoV-2 (G/GH/GR/S/L/V) were identified in the U.S. in Phase 1 (Figure 2A). However, the G-based clades (G/GH/GR), defined by the D614G mutation in the Spike (S) gene [35,36], accounted for >99% of sequences by Phase 2 (Figure S2). SARS-CoV-2 variants with the D614G mutation have been shown to be more infectious and exhibit some degree of resistance to certain monoclonal antibodies [37], yet they maintain convalescent serum neutralization sensitivity [38] and do not appear to worsen clinical outcomes (more on VOCs below) [39].

The aggregate average pair-wise distance (APD) among the G-based clades increased from 0.02% in Phase 1 to 0.06% in Phase 3, reflective of 2.3-, 3.0-, and 2.8-fold increases for clades G, GH, and GR, respectively (Figure 2A and Figure S3). These rates translate to 1.95-nt/month (clade G), 2.85-nt/month (clade GH), and 2.22-nt/month (clade GR) when expressed as the average numbers of changes observed in the viral genome each month (Figure S3A). For comparison, the overall APD measured for VOC lineages that emerged in 2021 (i.e., the Alpha, Beta, Gamma, Delta, and Epsilon) was between 0.02% and 0.09% (Figure S3B). As these genetic distances were comparable to those measured in the G-based clades in the U.S. in 2020, there is no reason to suspect that the VOCs will naturally accrue mutations more rapidly than non-variant lineages as the pandemic continues.

The observed increase in genetic diversity of SARS-CoV-2 in the U.S. in 2020 was due to an increase in the number of unique variants comprising clade G (+14%) and clade GR (+17%) (Figure 2A). However, despite the 3-fold increase in APD, clade GH had an overall decrease in the number of different variants from Phase 1 to Phase 3 (−11%) (Figure 2A). This finding suggests that the increase in genetic diversity in the GH clade resulted not from an increase in the number of variants but rather from the spread of fewer variants that were more divergent. This finding is further supported by observations of the numbers of mutations observed per sequence over time for each clade (Figure 2B). Specifically, whereas in Phase 1, clades G, GH, and GR averaged 7, 7, and 10 mutations/sequence, respectively, these frequencies increased by 1.7-, 2.4-, and 1.8-fold in Phase 3, another indication of a disproportionate increase in GH clade divergence. Similarly, panmixia, a metric indicating the degree to which random populations remain unstructured (i.e., non-divergent) over time [40], was calculated for the respective clades. We found there was significant divergence in both the G-based and S clades from Phase 1 to Phase 2 (*p <* 10^−6^), demonstrating that there was not random unstructured population mixing and that viral evolution was directional (suggesting selective pressures for some mutations) (Figure 2A).

Parallel analysis of the Alpha, Beta, Gamma, Delta, and Epsilon VOCs showed that these variants contained 26–36 mutations/sequences relative to their clade consensus, nearly twice the average observed among the G clades in 2020 (Figure 2B,C). These data suggest that the VOCs emerged via an atypical evolutionary pathway in which key mutations were acquired together within individuals (humans and/or other hosts) rather than only sequentially over the course of multiple person-to-person transmissions, further emphasizing the need for more targeted surveillance approaches.

### 3.3. Phylogenetic Analysis Reveals the Evolutionary Relationships of G Clades in the U.S. in 2020 to the VOCs

To determine the phylogenetic relationships of the G-based clades and VOCs in the U.S., we reconstructed a maximum-likelihood phylogenetic tree with a total of 1084 sequences from the U.S. (Figure S4). Though each of the G based clades formed well-defined groups, there was some intermingling observed, which may have been a result of mis-categorization at the time the clades were defined. The tree structure also reveals the evolutionary relationships between the VOCs and the clades from which they were derived. Specifically, the Alpha and Gamma VOCs branch within Clade GR, while the Beta and Epsilon are within Clade GH. For our analysis, Delta falls within Clade G since, at the time of collection, Clade GK had not yet been defined. This representation further highlights the greater genetic distance between VOCs, especially Alpha, Gamma, and Delta, relative to the G clades, again suggesting that the VOCs emerged through distinct evolutionary pathways.

### 3.4. SARS-CoV-2 Mutations Present in ≥5% of the Population Detected in the U.S. in 2020

Levels of SARS-CoV-2 sequencing in the U.S. determine both the sensitivity with which we are able to detect emerging mutations and our degree of statistical confidence that we have done or can do so. From U.S. viral sequences submitted to GISAID prior to 15 December 2020, we were able to detect mutations present in the population at frequencies ≥5% in Phases 1 and 2 and ≥14.5% in Phase 3 with 95% confidence (Figure S5). Increasing the sensitivity of and confidence in this type of analysis would require greater sampling, as is now being pursued. For instance, to detect mutations present at 1% or 0.1% frequency with 99% confidence, the viral sequencing capacity in the U.S. needs to increase by ~7.8-fold relative to 2020 levels (to 460 sequences/day) or ~78-fold (4600 sequences/day), respectively (Figure S5). Though these goals are now within reach in the U.S. due to increased funding for SARS-CoV-2 sequencing, achieving this level of surveillance worldwide, especially in regions with less abundant resources and higher population densities, seems unlikely, and more practical alternatives must be considered. This approach is in agreement with a recommendation by the European Centre for Disease Prevention and Control advocating for targeted sequencing of SARS-CoV-2 cases in select populations (e.g., vaccine breakthrough and outbreak clusters) as a complement to broader surveillance [41].

### 3.5. At Least 54 New Amino Acid Changes Emerged in 2020 and Persisted in ≥5% of the U.S. Population in at Least one Phase of the Spread

**Figure 3:**
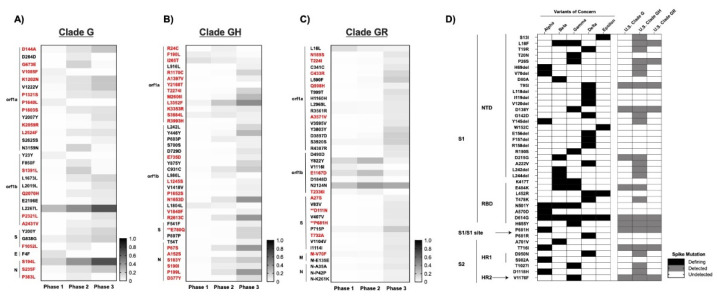
Emerged mutations in SARS-CoV-2 have increased in the U.S: (**A**–**C**) Non-clade-defining mutations of Clades G, GH, and GR. Emerging mutations are shown in which, during at least one Phase in 2020, the frequency exceeded 5%. Sequences were compared to the majority-consensus sequence for each respective clade in Phase 1. Mutation designations reflect the relative amino acid positions in the gene regions. There were no common mutations among the G-based clades. Red text denotes non-synonymous mutations. (**) Mutations that occur in T- or B-cell epitope regions. (**D**) Comparison of non-synonymous mutations and deletions in Spike between VOCs and the U.S. G-based clades across all three phases. The presence or absence of mutations is indicated by shading: VOC-defining mutations (black), mutations detected during at least one phase in the respective G-clade (gray), and undetected in either VOC or U.S. G-clades (white).

To detect the emergence of new SARS-CoV-2 mutations in the U.S. in 2020, majority-rule consensus sequences of each G clade from Phase 1 were compared to all respective sequences in the subsequent two phases (Tables S3–S5). Only mutations present in ≥5% of the population were included in the analyses for reasons described in Materials and Methods, although select mutations of specific interest present at lower frequencies were also noted and are discussed below. About half of the non-clade-defining mutations that arose in the U.S. in 2020 and persisted at frequencies ≥5% were nonsynonymous, i.e., clade G: ORF1a (9 nonsynonymous mutations), ORF1b (4), Spike (1), Nucleocapsid (3); clade GH: ORF1a (12), ORF1b (6), Spike (1), Nucleocapsid (6); and clade GR: ORF1a (6), ORF1b (3), Spike (4), Matrix (1). While some mutations arose and then declined during 2020 (or shifted geographically), most persisted and increased in frequency (Figure 3A–C). In particular, clade G mutation N^S194L^ and clade GH mutations ORF1a^L3352F^ and ORF1b^N1653D;R2613C^ increased more than 40% from Phase 1 to Phase 3. Many new mutations were detected in Phase 3 despite the limitations of extremely shallowing sampling.

The ratio of nonsynonymous-to-synonymous fixation rates (dN/dS) serves to indicate the nature of selective pressure at individual coding positions in a gene. More specifically, this ratio can suggest neutral (dN/dS~1), negative/purifying (dN/dS < 1), or positive (dN/dS > 1) selection at a given site. The overall dN/dS for SARS-CoV-2 has been shown to be under negative selection [36]; however, we did observe some mutations in Spike that were under positive selection (dN/dS > 1). Several of these mapped to T- and B-cell epitopes, including clade G Nucleocapsid^S235F^, clade GH S^E780Q^, and clade GR S^D111N;P681H^ (Figure S6). Some positions in predicted antibody epitopes in Spike, e.g., S^94;562;816^, were found to be under purifying selection (dN/dS < 1). The same is true of the alanine residue in the furin cleavage site of Spike (RR**A**R), suggesting that substitution at this position confers a replicative disadvantage. This conclusion is supported by in vitro experiments wherein virus containing an RR**K**R polybasic cleavage site produces larger syncytia but exhibits decreased infectivity [42]. Conversely, the proline adjacent to the S1/S2 cleavage site (S^681^) required for infection in human lungs [42,43,44] was under positive selection, indicative of room for evolutionary improvement. S^5^, located 5′ to the start of the NTD/S1, was also under positive selection in all G-based clades. Outside of Spike, we identified several positively selective nonsynonymous changes in both B-cell and MHC-Class I/II T-cell epitopes in Nucleocapsid (Figure S7).

In accordance with an elevated level of importance, VOCs are defined primarily, though not exclusively, by their associated Spike mutations in functionally and immunogenically important regions within the N-terminal and Receptor Binding Domains [45]. Critical investigations have been and are being conducted to determine how emergent Spike mutations affect vaccine efficacy [46,47], immunotherapeutics (i.e., monoclonal antibodies and convalescent plasma) [3,47], and viral transmission and pathogenesis [35,39,48]. Selection of Spike mutations among recipients of convalescent plasma is particularly concerning [48,49,50], since the partial protection conferred by such treatments is likely conducive to immune escape, and may have contributed to the emergence of the Alpha, Beta, Gamma, Delta, and Epsilon VOCs, and, most recently, the Omicron VOC [51]. 

### 3.6. Mutations in Spike and Implications for COVID-19 Vaccines

Since all current vaccines were designed to potentiate cellular and humoral immune memory responses against the SARS-CoV-2 Spike protein [52], the emergence of mutations in the viral gene encoding Spike warrant special attention, including those found at overall frequencies of less than 5% in all three phases. For instance, three mutations in known B-cell epitopes in Spike became prominent in Clades GH and GR starting in Phase 2 (Supplementary Materials Figure S6). The frequency of these mutations increased with each new phase of infections, perhaps constituting a rare example of enrichment due to immune resistance outside of VOCs or due to founder effects. It has been shown in studies using the Ad26.COV2.S (Janssen/Johnson & Johnson) vaccine that several VOCs (Alpha, Beta, and Gamma) produced similar CD4 and CD8 responses; however, there were reduced neutralizing antibody responses against the Beta and Gamma VOCs among vaccine recipients [53]. In contrast, studies in nonhuman primates vaccinated with mRNA-1273 (Moderna) showed measurable dose–responses of circulating and mucosal antibodies against the VOCs [54]. Administration of either BNT162b2 (Pfizer/BioNTech) or ChAdOx1 nCOV-19 (Oxford/AstraZeneca) generated neutralizing antibodies against the Delta VOC but with 3-to-5-fold lower titers compared to the Alpha VOC, as well as a modestly decreased vaccine effectiveness in Delta vs. Alpha [55,56]. Although the VOCs were not detected in the U.S. until late 2020 or early 2021, our analysis shows that many of their individual defining Spike mutations were present in the U.S. as early as Phase 1 (Figure 3D). However, with the exception of S^P681H^, the average frequencies of these individual mutations were all <1%. S^E484K^ and S^N501Y^ mutations are present in multiple VOCs and are particularly concerning, having been reported to decrease susceptibility to antibody neutralization [3]. Moreover, neutralizing antibodies from COVID-19 patients or SARS-CoV-2-infected humanized mice were shown to be less effective against Alpha, Beta, and Gamma VOCs, each of which harbor the S^E484K^ and/or S^N501Y^ mutations [57], although immunity induced by mRNA-based vaccines appears to remain at least partially effective against E484K-containing VOCs [58,59,60,61]. With respect to the durability of vaccine immunity, antibody functionality persists at low levels for at least 6 months after primary mRNA-1273 vaccination when compared to the VOCs. However, a greater reduction in antibody recognition was observed in cases of the Gamma VOC, of which the efficacy for the BNT162b2, Ad26.COV2.S, and mRNA-1273 vaccines has been studied against the VOCs as well as support booster vaccinations to prolong protection [11,56,62,63,64,65,66,67]. Comparably, sera from individuals previously infected with the Beta or Gamma VOCs were less effective in neutralizing the Delta VOC than from a strain isolated from Australia early in the pandemic [68].

Fortunately, the evolutionary distance between these SARS-CoV-2 VOCs and complete resistance to adaptive immunity conferred by current vaccines remains substantial. Not only does vaccine-induced immunity remain highly effective against hospitalization and death caused by any of the current VOCs [69], but the sudden worldwide dominance of the Delta VOC in 2021 was due almost completely to its increased transmission and not vaccine resistance. Whether this will also prove to be true for more recently discovered VOC, Omicron, remains to be seen, since the relative transmissibility, vaccine resistance, and pathogenicity of this variant have yet to be fully characterized. However, the more than 30 Spike gene mutations in the Omicron VOC relative to the Wuhan-Hu-1 reference are again suggestive of an evolutionary environment distinct from the one most prevalent in the U.S. in 2020.

Although multiple variants harboring individual mutations thought to incrementally contribute to immune resistance were found in the U.S. at low frequencies early in 2020, our analysis indicates that, after nearly a year, any selective advantage conferred by these mutations was insufficient to significantly expand their representation in the viral population. It is therefore perhaps not surprising that, after analyzing tens of thousands of sequences representing millions of cases in the U.S. in 2020, we found no evidence of variants harboring multiple immune resistance mutations serially acquired over the course of several viral transmissions among the sequences analyzed. Of course, our analysis is not necessarily predictive of what will occur in the future with the now-dominant Omicron VOC, or newly emergent VOCs in the face of rising immune pressure due to prior infection and/or vaccination. Our work will, however, serve as an important baseline for comparison in the event of changes in the rate or mode of evolution of SARS-CoV-2.

## 4. Conclusions

Although the replication fidelity of SARS-CoV-2 is quite high relative to other RNA viruses [5,70], its genetic stability is countered by the expansive spread of the virus both in the U.S. and globally. On balance, evolution of this virus might be best characterized as slow but inexorable, driven largely by genetic drift but also influenced by selective pressures such as relative infectivity, relative transmissibility, and to a lesser extent, immune evasion. In contrast, the relatively recent and suddenly emergent VOCs are characterized by a significantly higher degree of genetic divergence that is rapidly acquired and clearly confers a replicative advantage. These variants were most likely the product of isolated cases in which the evolutionary environments differ substantially from the norm; e.g., from chronically infected immunosuppressed individuals, including those who received treatment with monoclonal antibodies or convalescent serum [49,50,51], and/or animal reservoirs.

Regardless of the evolutionary pathways taken, the emergence of more rapidly transmissible and partially immune resistant variants in both the U.S. and global viral populations threatens the continued efficacy of current treatments, and vaccines and will do so increasingly as the virus continues to spread and evolve. The sudden worldwide dominance of the Delta VOC in 2021, and the more recent emergence and spread of the Omicron VOC, exemplifies this concern. In response, scientists worldwide are coming together to increase timely SARS-CoV-2 sequencing and analysis to help guide decisions on current and future health policies. In addition, because it is becoming increasingly apparent that the current VOCs emerged from atypical evolutionary environments, targeted worldwide surveillance of high-risk human infections and animal reservoirs may be the best means of detecting future VOCs as they emerge, particularly in resource-limited regions.

In 2020, efforts to slow the spread of the virus within the U.S. were hampered by incomplete adherence to recommended preventative measures (e.g., mask wearing, hand washing, social distancing) as well as inconsistent and even conflicting messaging regarding these measures. These failures indirectly accelerated viral divergence, increased genetic diversity, and resulted in the accumulation of nonsynonymous mutations, many of which are within epitopes now associated with resistance to neutralizing antibodies and that may ultimately contribute to immune evasion. Now, in 2022, the Delta VOC rapidly spread across the U.S. as well as Omicron VOC, primarily among the unvaccinated population but also, to a lesser degree, among the vaccinated, raising the bar for herd immunity even higher.

As more people become infected and/or are vaccinated, selective pressure for immune-resistant variants will increase concomitantly. It is therefore essential that we monitor individuals who become infected post-vaccination, as well as those with prolonged infection (e.g., immunocompromised individuals), both to better understand the capacity of SARS-CoV-2 for escape from vaccine-induced immunity and to identify and isolate resistant variants early when they emerge. Finally, although our study focused on the U.S., we recognize that the emergence of potential new VOCs and variants of interest is an ongoing and global concern. We must all therefore diligently and intelligently use our resources to detect and analyze new variants and, just as importantly, continue to encourage measures to prevent their spread.

## Data Availability

All data are available in the main text or the Supplementary Materials.

## Supplementary Materials

The following are available online at https://www.mdpi.com/article/10.3390/v14010104/s1, Figure S1: SARS-CoV-2 epidemic at the divisional level in the U.S., Figure S2: Proportion of SARS-CoV-2 genomes in each GISAID clade, Figure S3: Genetic distance over time in 2020 for GISAID clades and Variants of Concern, Figure S4 Phylogenetic analysis of SARS-CoV-2 in the U.S., Figure S5: Determining number of sequences of sampling at a given probability to detect variant frequency, Figure S6: Mutation in G clades that are located in MHC-II HLA-DR T-cell and B-cell epitopes, Figure S7: Selection pressure and epitopes detected in Nucleocapsid, Table S1: Demographics and Regional Division based on the U.S. Census Bureau, Table S2: SARS-CoV-2 T-cell and B-cell epitopes Table S3: Non-associated Clade G mutations that either persisted or emerged during 2020, Table S4: Non-associated Clade GH mutations that either persisted or emerged during 2020, Table S5: Non-associated Clade GR mutations that either persisted or emerged during 2020, Supplemental Dataset: Sequences Analyzed.
