# Supplementary material for: A Pre-Vaccination Baseline of SARS-CoV-2 Genetic Surveillance and Diversity in the United States
Source: Viruses. 2022 Jan 7;14(1):104. doi: 10.3390/v14010104 (PMC8778900; doi:10.3390/v14010104)
Supplement: Supplementary file 1 [file viruses-14-00104-s001.zip › viruses-1536673-supplementary-update.pdf]

Supplementary Material

A pre-vaccination baseline of SARS-CoV-2 genetic surveillance and diversity in the United States

Adam A. Capoferri [1,2,*], Wei Shao [3], Jon Spindler [1], John M. Coffin [4], Jason W. Rausch [1], and Mary F. Kearney [1]

[1] HIV Dynamics and Replication Program, Center for Cancer Research, NCI-Frederick, Frederick, MD 21702, USA; adam.capoferri@nih.gov (A.A.C.); jspindler@mail.nih.gov (J.S.); rauschj@mail.nih.gov (J.W.R.); kearneym@mail.nih.gov (M.F.K.)
[2] Department of Microbiology and Immunology, Georgetown University, Washington DC 20007, USA; ajl160@georgetown.edu
[3] Advanced Biomedical Computing Science, Frederick National Laboratory for Cancer Research, Frederick, MD 21702, USA; shaow@mail.nih.gov
[4] Department of Molecular Biology and Microbiology, Tufts University, Boston, MA 02129, USA; john.coffin@tufts.edu
* Correspondence: adam.capoferri@nih.gov

This file includes:

Supplemental Figures. S1 to S7
Supplemental Tables S1 to S5
References 71-75

Supplemental Figures and Tables

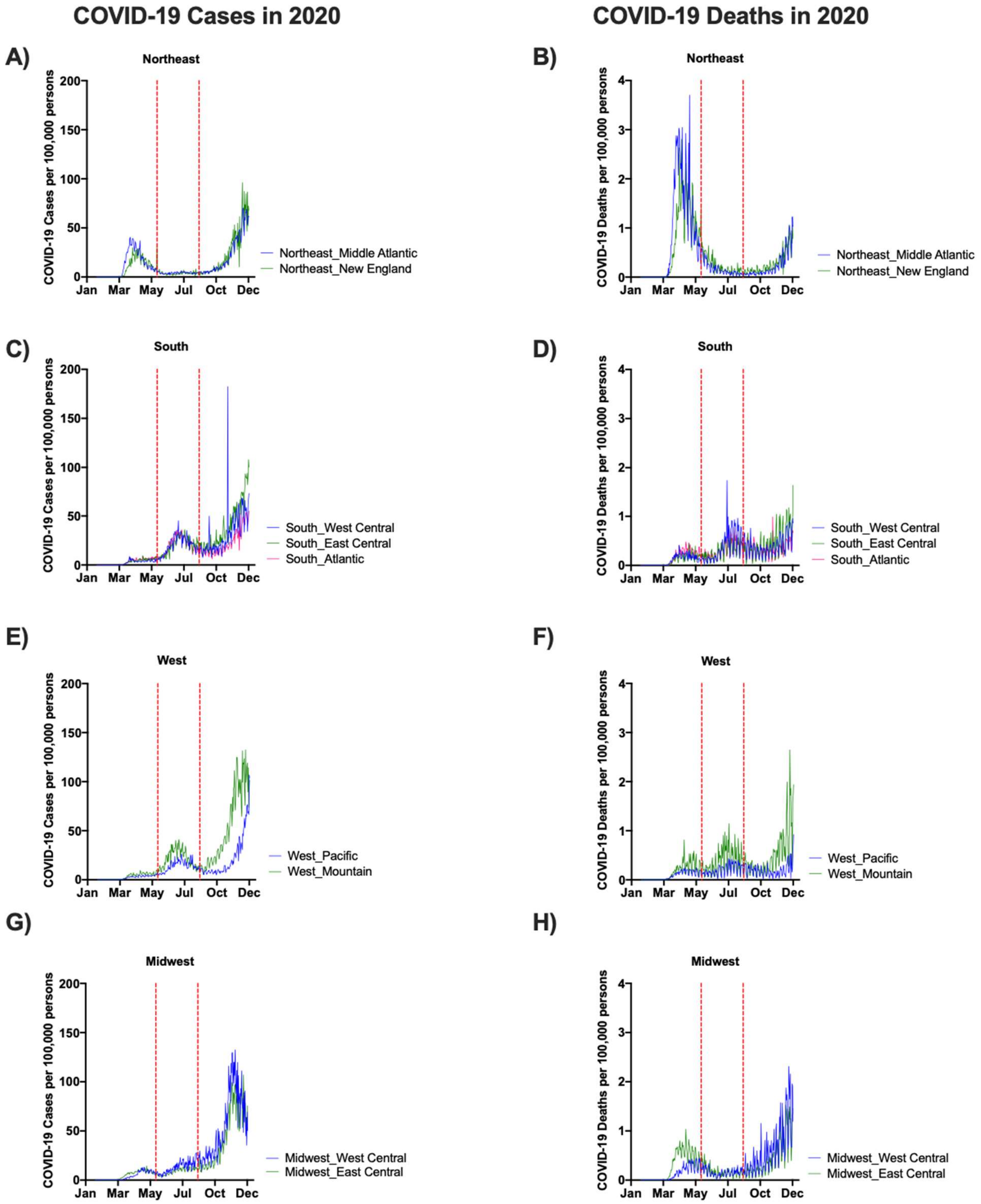

Supplemental Figure S1

SARS-CoV-2 epidemic at the divisional level in the U.S. (**A, C, E, G**) Number of daily COVID-19 Cases in 2020 per 100,000 persons normalized to the 2019 estimated population in each sub-region. (**B, D, F, H**)

Number of daily COVID-19 Deaths in 2020 per 100,000 persons normalized to the 2019 estimated population in each sub-region. (**A-B**) Northeast divisions of Middle Atlantic and New England. (**C-D**) Southern divisions of West Central, East Central, and Atlantic. (**E-F**) Western divisions of the Pacific and Mountain. (**G-H**) Midwestern divisions of West Central and East Central. Each division is colored respectively with dotted red lines indicating the separation of Phases. Population and date of first COVID-19 case in each sub-region is report in **Supplemental Table S1**.

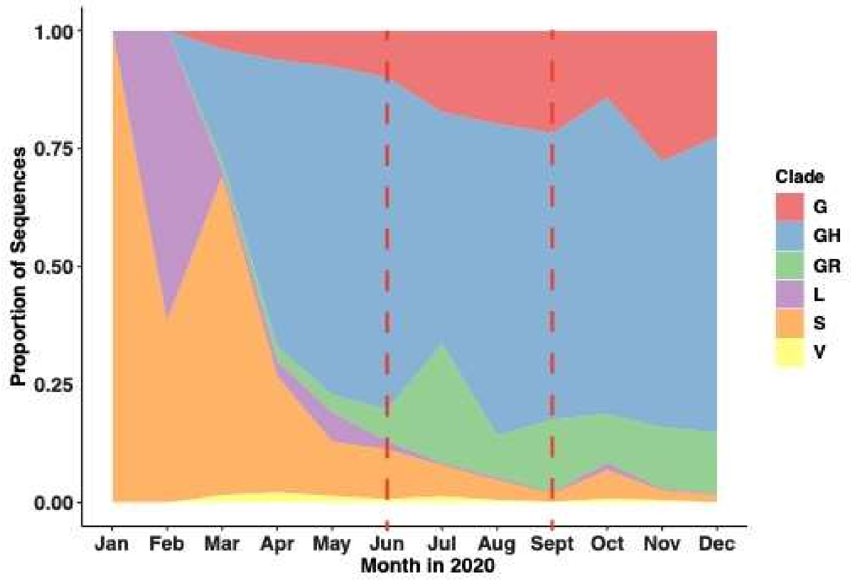

Supplemental Figure S2

Proportion of SARS-CoV-2 genomes in each GISAID clade. The proportion of sequences observed during each month in 2020 based on the GISAID assigned Clades was calculated. The dotted red lines indicate the separation of Phases 1, 2, and 3. Data were accessed 18-December-2020 whereby any sequences submitted or collected by 15-December-2020 were considered. Clades G, GH, and GR represented the majority of all sequences by Phase 3.

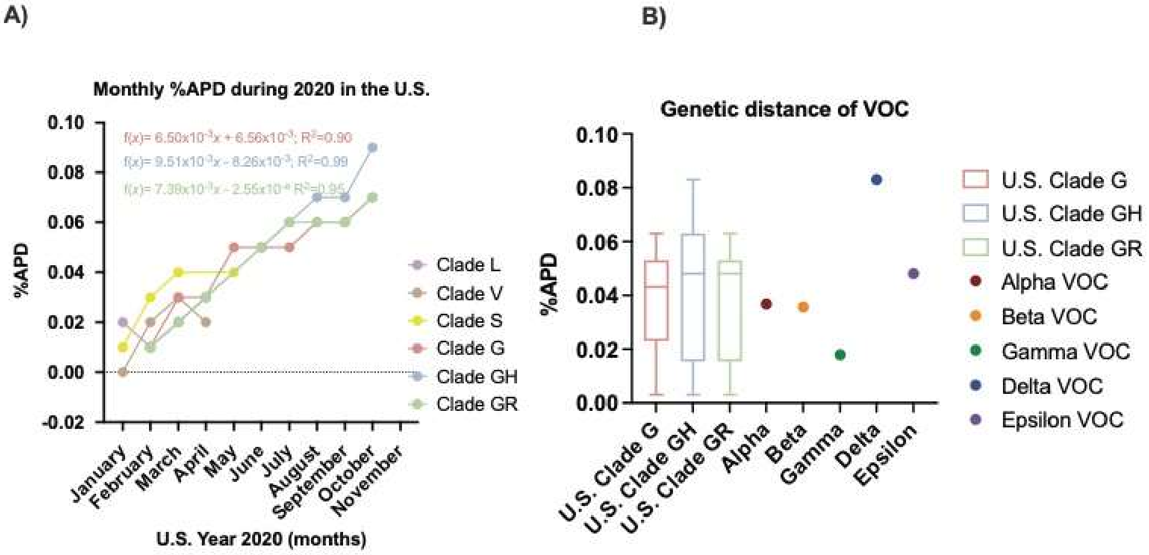

Supplemental Figure S3

Genetic distance over time in 2020 for GISAID clades and Variants of Concern. (**A**) Average pair-wise distance (APD) was calculated per month for sequences within each clade. Time points with less than 10 sequences were excluded. Three randomized subsamples of 50 sequences each were analyzed and the APD was calculated to ensure consistency between each subsampling. If there were 11-50 sequences for a clade at a given month, no subsampling was performed. A standard linear regression was run for clades G, GH, and GR. The rate of change in %APD over time is noted in the figure with the goodness-of-fit (R^2) reported. The %APD was plotted according to each respective month. The rate of change was $6.50x10^{-3}$ APD/month (clade G), $9.51x10^{-3}$ APD/month (clade GH), and $7.39x10^{-3}$ APD/month (clade GR); which corresponded to 1.95 nt/month (clade G), 2.85 nt/month (clade GH), and 2.22 nt/month (clade GR) based on the SARS-CoV-2 genome of ~30,000 bp. (**B**) Comparison of the %APD between the VOCs and the G-based Clades. A box and whister plot of the %APD of the G-based clades in the U.S. during 2020 is shown. The median (solid line) is marked within the interquartile range, with the whiskers as the minimum and maximum. The VOC sequences sampled in the U.S. were accessed through GISAID.org with the collection date between 1-November-2020 to 31-March-2021. For the Delta VOC, all U.S. high quality sequences deposited until 27-July-2021 were selected. Total APD was calculated for the VOCs with a randomized subsampling of 2,500 sequences for the Delta VOC.

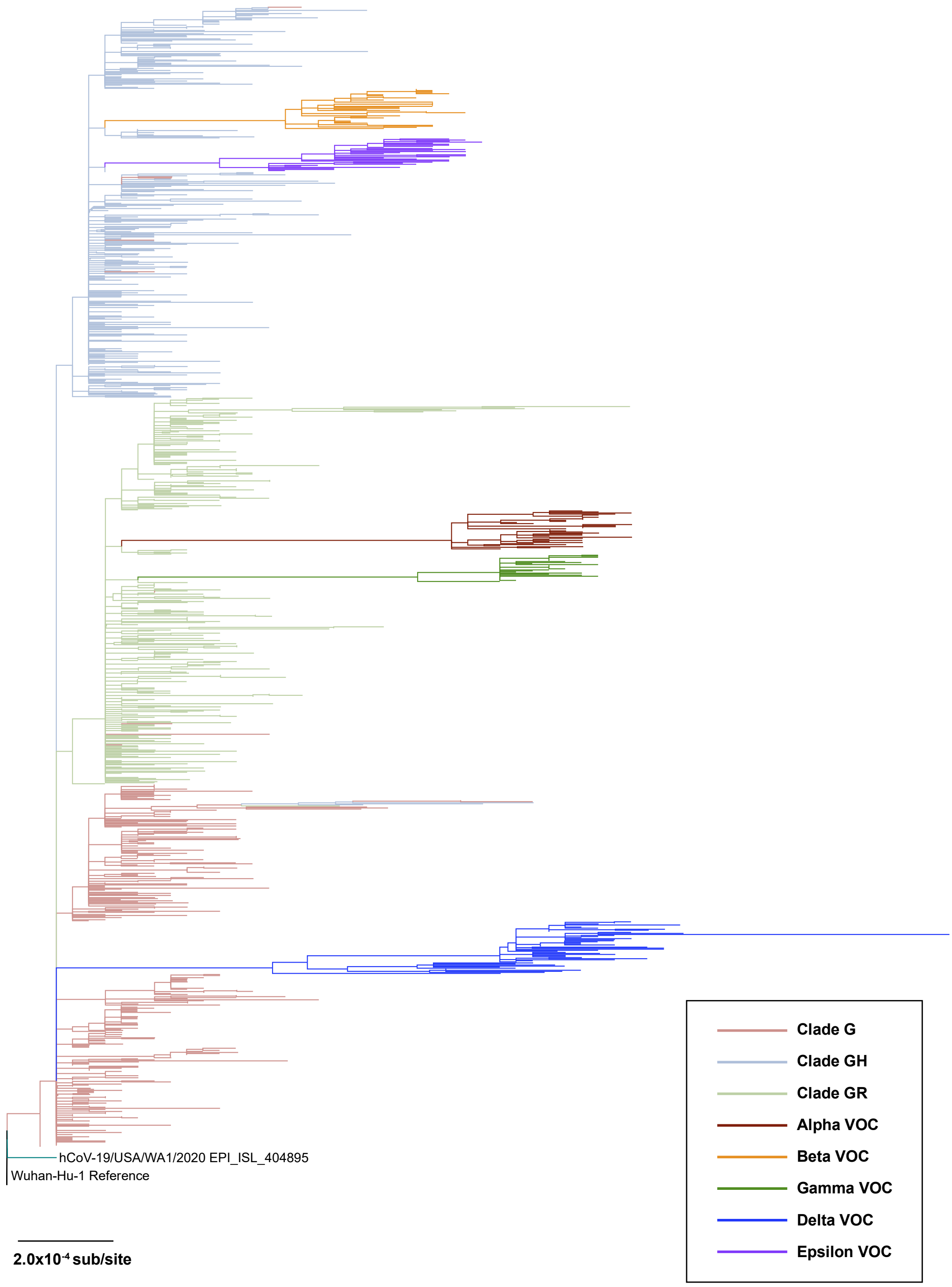

Supplemental Figure S4

Phylogenetic analysis of SARS-CoV-2 in the U.S. Maximum-likelihood phylogeny of U.S. and reference sequences. The phylogeny is rooted on the Wuhan-Hu-1 Reference genome and includes the first collected

U.S. SARS-CoV-2 genome (USA/WA1/2020 EPI_ISL_404895). Branches are colored according to either their associated GISAID Clade nomenclature or by VOC. The scale is set to $2.0x10^{-4}$ substitutions/site.

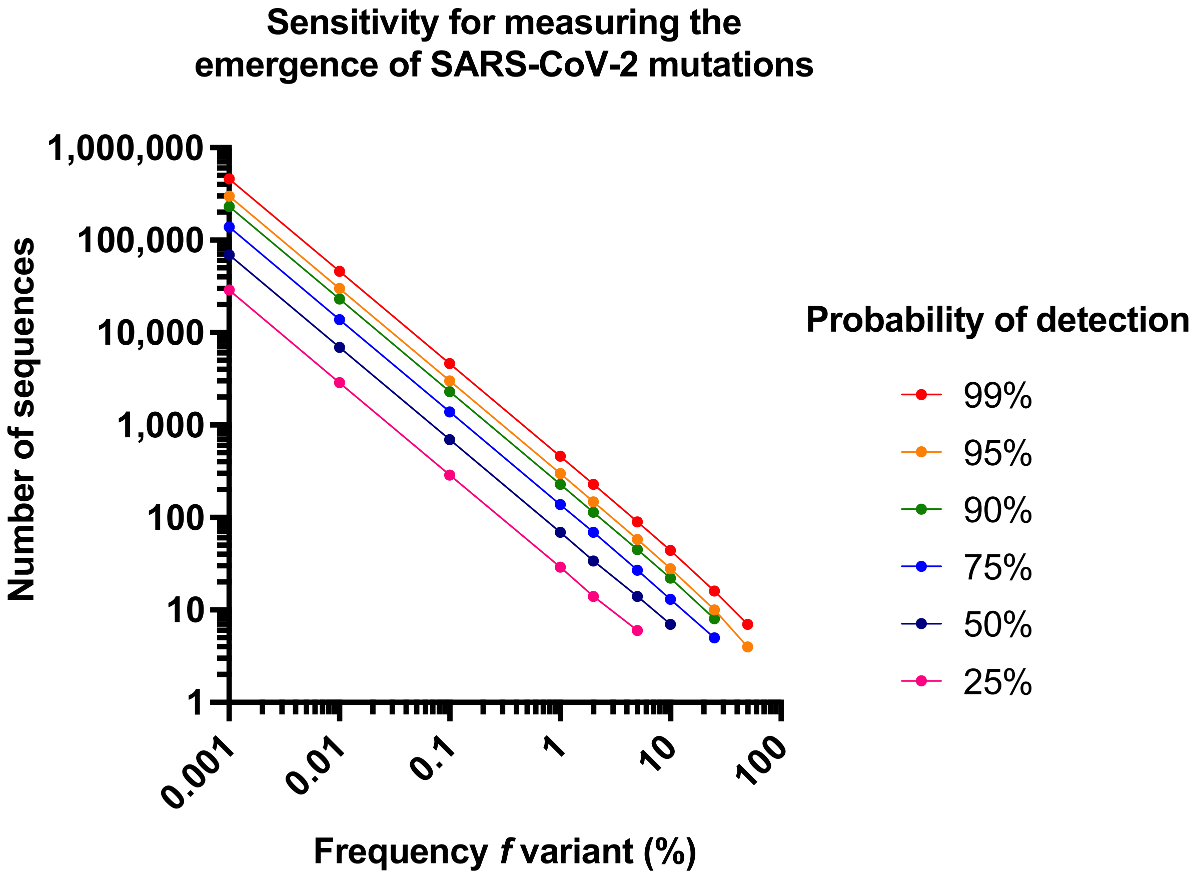

Supplemental Figure S5

Determining number of sequences of sampling at a given probability to detect variant frequency. To determine the number of sequences required to detect at a certain mutation frequency with a given probability, follows the quotient: $N = \frac{\log(1-p)}{\log(1-f)}$. Where ($N$) is the number of sequences of any given sample that is required to be sampled to determine (f), the frequency the mutant detected, at a given (p), the probability of detecting the mutation at the provided frequency.

Clade	Gene (amino acid position)	Phase	Epitope	U.S. Frequency	Description of epitope
G	N (221-235)		L L L L D R L N Q L E S K M S		T-cell MHC-II HLA-DR
		1	. . . . F	1.58%	
		2	. . . . F	11.29%	
		3	. . . . F	22.01%	
GH	S (776-785)		A L T G I A V E Q D K N T Q E F A Q V		IgG/IgA and B-cell antibody
		1	. . . .	0%	
		2	. . . . Q	0.04%	
		3	. . . . Q	15.98%	
GR	S (110-115)		L D S K T Q		B-cell antibody
		1	. . . . . .	0%	
		2	. N	0.12%	
		3	. N	8.75%	
	S (673-684)		Y Q T Q T N S S P R R A R		B-cell antibody
		1	. . . .	0%	
		2	. . . . H	0.12%	
		3	. . . . H	7.50%	

Supplemental Figure S6

Mutations in G clades that are located in MHC-II HLA-DR T-cell and B-cell epitopes. Nucleocapsid MHC-I/II T-cell and Spike B-cell epitopes with detected mutations are shown. Nonsynonymous mutations were detected in Clades GH and GR starting in Phase 2 through Phase 3 (highlighted). The amino acid position of the epitope is denoted next to N (Nucleocapsid) and S (Spike). The U.S. frequency of detection relative to the Clade is noted for each Phase. Epitopes examined are defined by **Supplemental Table S2.**

Mutation	G			GH			GR			Restriction if known	Epitope
	1	2	3	1	2	3	1	2	3		
A35A	<1	<1	<1	>1	<1		<1	<1	<1		
P42P		<1						<1	<1		
T43T		>1			>1						
P67S		>1		>1	>1	>1					
A152S					<1	>1		>1			
S183Y				>1	>1	>1				B-cell	SRGGSQA**S**SRSSSRSRNSSRNSTPGSSRGTS
S190I		>1			>1	>1	>1	>1	>1	B-cell	SRGGSQASSRSS**S**RSRNSSRNSTPGSSRGTS
S194L		<1	>1	>1	>1	>1				B-cell	SRGGSQASSRSSSRSRNS**S**RNSTPGSSRGTS
P199L			>1	>1	>1	>1		>1		B-cell	SRGGSQASSRSSSRSRNSSRNST**P**GSSRGTS
S235F	>1		>1	>1	>1	>1				MHC-II	LLLLDRLNQLESKM**S**
K261K						<1		<1	<1	B-cell<br>MHC-I	KSAAEASKKPRQ**K**RT<br>**K**RTATKAYNVTQAFGRRGP
D377Y	>1		>1	>1	>1	>1	>1	>1		MHC-II	FPPTEPKKDKKKKA**D**ET
P383F	>1		<1	<1	<1	<1	<1	<1			

Supplemental Figure S7. Selection pressure and epitopes detected in Nucleocapsid. Mutations detected ≥5% in Nucleocapsid are reported with non-synonymous changes in red. Selection pressure measured by dN/dS across positions in Nucleocapsid for each clade and phase are noted. B-cell antibody and MHC-I/II T-cell epitopes were examined. MHC-II (HLA-DR) allotypes included: DRB*01:01, DRB*03:01, DRB*04:01, DRB*07:01, DRB*11:01, and DRB*15:01. Amino acid residues within epitopes that are bold and underlined indicate where the mutation is located within the mapped epitope region.

Supplemental Table S1

Demographics and Regional Division based on the U.S. Census Bureau. The 2019 estimated residential population for each State is reported. The first reported confirmed plus probable cases of COVID-19 are included (https://covidtracking.com/). The U.S. is generally divided into the Northeast, South, West, and Midwest. Where each major region can be divided into sub-regions. These estimated populations were then used to normalize the incidence of COVID-19 cases and deaths in the U.S. at the sub-regional level.

U.S. Region	U.S. Sub-region	State	Estimated Resident Population (2019)	Date of first reporting cases of COVID-19
Northeast (55,982,803)	Middle Atlantic (41,137,740)	NJ	8,882,190	05-March-2020
		NY	19,453,561	03-March-2020
		PA	12,801,989	06-March-2020
	New England (14,845,063)	CT	3,565,287	08-March-2020
		ME	1,344,212	12-March-2020
		MA	6,892,503	04-March-2020
		NH	1,359,711	04-March-2020
		RI	1,059,361	01-March-2020
		VT	623,989	08-March-2020
South (125,580,448)	West Central (40,619,450)	AR	3,017,804	12-March-2020
		LA	4,648,794	09-March-2020
		OK	3,956,971	07-March-2020
		TX	28,995,881	06-March-2020
	East Central (19,176,181)	AL	4,903,185	13-March-2020
		KY	4,467,673	07-March-2020
		MS	2,976,149	12-March-2020
		TN	6,829,174	05-March-2020
	Atlantic (65,784,817)	DE	973,764	11-March-2020
		FL	21,477,737	03-March-2020
		GA	10,617,423	04-March-2020
		MD	6,045,680	06-March-2020
		NC	10,488,084	04-March-2020
		SC	5,148,714	07-March-2020
		VA	8,535,519	08-March-2020
		D.C.	705,749	08-March-2020
		WV	1,792,147	18-March-2020
West (72,465,722)	Pacific (53,492,270)	AK	731,545	17-March-2020
		CA	39,512,223	04-March-2020
		HI	1,415,872	07-March-2020
		OR	4,217,737	05-March-2020
		WA	7,614,893	19-January-2020
	Mountain (18,973,452)	AZ	7,278,717	04-March-2020
		CO	5,758,736	04-March-2020
		ID	1,787,065	14-March-2020
		MT	1,068,778	12-March-2020
		NV	3,080,156	05-March-2020
		NM	2,096,829	12-March-2020
		UT	3,205,958	07-March-2020
		WY	578,759	12-March-2020
Midwest (66,682,283)	West Central (19,779,852)	IA	3,155,070	09-March-2020
		KS	2,913,314	08-March-2020
		MN	5,639,632	06-March-2020
		MO	6,137,428	08-March-2020
		NE	1,934,408	07-March-2020
		ND	762,062	12-March-2020
		SD	884,659	11-March-2020
	East Central (46,902,431)	IL	12,671,821	04-March-2020
		IN	6,732,219	06-March-2020
		MI	9,986,857	01-March-2020
		OH	11,689,100	09-March-2020
		WI	5,822,434	04-March-2020

Supplemental Table S2

SARS-CoV-2 T-cell and B-cell epitopes in Spike and Nucleocapsid. Epitopes demonstrating cross-reactivity other human coronaviruses are denoted (#). In some studies, specific HLA alleles were examined for MHC-I/II. MHC-II HLA-DR allotypes included: DRB*01:01, DRB*03:01, DRB*04:01, DRB*07:01, DRB*11:01, and DRB*15:01 ($)

Epitope	Location	Restriction	Reference
MKDLSPRWYFYYLGTGPEAG	Nucleocapsid	MHC-II	[71, 72]
SKLWAQCVQLHNDIL	Nsp7	MHC-II	
HNDILLAKDTTEAFE	Nsp7	MHC-I	
MEVTPSGTWL	Nucleocapsid	MHC-I (HLA-B*40)	
LTDEMIAQY	Spike	MHC-I (HLA-A*01)	[72]
KTFPPTEPKK	Nucleocapsid	MHC-I (HLA-A*03)	
ATEGALNTPK	Nucleocapsid	MHC-I (HLA-A*03)	
QYIKWPWYI	Spike	MHC-I (HLA-A*24)	
KDGIIWVATEGALNT	Nucleocapsid	MHC-II (HLA-DR$#)	
GTWLTYTGAIKLDDK	Nucleocapsid	MHC-II (HLA-DR$#)	
RWYFYYLGTGPEAGL	Nucleocapsid	MHC-II (HLA-DR$#)	
ASWFTALTQHGKEDL	Nucleocapsid	MHC-II (HLA-DR$#)	
ASAFFGMSRIGMEVT	Nucleocapsid	MHC-II (HLA-DR$#)	
IGYYRRATRRIRGGD	Nucleocapsid	MHC-II (HLA-DR$#)	
LLLLDRLNQLESKMS	Nucleocapsid	MHC-II (HLA-DR$#)	
ITRFQTLLALHRSYL	Spike	MHC-II (HLA-DR$#)	
KYFKNHTSP	Spike	B-cell	[73]
TTKR	Spike	B-cell	
YYHKNNKSWM	Spike	B-cell	
ASTEK	Spike	B-cell	
AWNRKR	Spike	B-cell	
EQDKNTQ	Spike	B-cell	
GTNTSN	Spike	B-cell	
KYNENGT	Spike	B-cell	
LDSKTQ	Spike	B-cell	
PKKS	Spike	B-cell	
YQTQTNSPRRAR	Spike	B-cell	
VLTESNNKFLPFQQFGRDIA	Spike	B-cell	[74]
KPSKRSFIEDLLFNKVTLAD	Spike	B-cell	
ELDSFKEELDKYFKNHTSPD	Spike	B-cell	
SDSTGSNQNGER	Nucleocapsid	B-cell	[75]
TNSSPDD	Nucleocapsid	B-cell	
SRGGSQASSRSSSRSRNSSRNSTPGSSRGTS	Nucleocapsid	B-cell	
SGKGQQQQG	Nucleocapsid	B-cell	
KSAAESKKPPQKRT	Nucleocapsid	B-cell	
FPPTEPKKDKKKKADET	Nucleocapsid	B-cell	
GDGKMKDLSPRWYFYYLGTGPEAGLPYGANKD GIIWVATEG	Nucleocapsid	MHC-I	
RMAGNGGDAALALLLLDRLNQLES	Nucleocapsid	MHC-I	
KRTATKAYNVTQAFGRRGP	Nucleocapsid	MHC-I	
QFAPSASAFFGMSRIGMEVTPSGTWLTYTG	Nucleocapsid	MHC-I	
GGDGKMKDLSPRWYFYYLGTGPEAGLPYGAN K	Nucleocapsid	MHC-II	
NGGDAALALLLLDRLNQLESKMSGKG	Nucleocapsid	MHC-II	
RQGTDYKHWPQIAQFAPSASAFFGMSRI	Nucleocapsid	MHC-II	

Supplemental Table S3.

Non-associated Clade G mutations that either persisted or emerged during 2020. Data were extracted where, during at least one Phase period, the frequency of a particular mutation was ≥5% compared to the clade Phase 1 majority consensus. The mutation specifies the nucleotide change as well as the amino acid change with coordinates of the gene and its product. Non-synonymous mutations are shown in red.

Gene	Mutation	Frequency (%)			Net Change (%)
		Phase 1	Phase 2	Phase 3	Phase 1→3
orf1a	a431c(D144A)	1.72	11.29	21.81	+20.09
	c792t(D264D)	0	7.59	0	0
	g2017a(G673E)	0	16.83	1.54	+1.54
	g3253t(V1085F)	5.1	0.07	0	-5.10
	g3606t(K1202N)	3.21	11.29	21.81	+18.60
	t3666c(V1222V)	1.54	11.04	21.62	+20.08
	c3961t(P1321S)	0.40	8.68	11	+10.60
	c4919t(P1640L)	6.02	0	0	-6.02
	c5407t(P1803S)	0.53	8.82	10.62	+10.09
	c6020t(Y2007Y)	0	1.24	11.20	+11.20
	a6176g(K2059R)	0	0.49	5.02	+5.02
	a7572c(L2524F)	0.40	8.57	10.23	+9.83
	c7875t(S2625S)	0.09	0.49	5.02	+4.93
	t9477c(N3159N)	0.13	17.29	1.54	+1.41
orf1b	c69t(Y23Y)	5.01	0.21	0.39	-4.62
	c2550t(F850F)	0.31	9.03	1.35	+1.04
	c4172t(S1391L)	0.40	18.38	1.54	+1.14
	c5019t(L1673L)	0.04	8.54	15.83	+15.79
	c6057t(L2019L)	0	1.2	10.81	+10.81
	g6210t(Q2070H)	0	6.99	7.53	+7.53
	a6588g(E2196E)	0	1.09	6.76	+6.76
	a6801g(L2267L)	25.99	43.9	67.37	+41.38
	c6962t(P2321L)	0	1.13	10.81	+10.81
	c7292t(A2431V)	0	5.75	7.14	+7.14
S	t600c(Y200Y)	0	8.57	15.83	+15.83
	t2514c(G838G)	5.5	12.14	18.15	+12.65
	c3156a(F1052L)	0.09	6.67	0	-0.09
E	c12t(F4F)	10.82	0.18	0	-10.82
N	c581t(S194L)	17.19	41.78	68.34	+51.15
	c704t(S235F)	1.58	11.29	22.01	+20.43
	c1148t(P383L)	0.13	6.81	0.19	+0.06

Supplemental Table S4.

Non-associated Clade GH mutations that either persisted or emerged during 2020. Data were extracted where, during at least one Phase period, the frequency of a particular mutation was ≥5% compared to the clade Phase 1 majority consensus. The mutation specifies the nucleotide change as well as the amino acid change with coordinates of the gene and its product. Non-synonymous mutations are shown in red.

Gene	Mutation	Frequency (%)			Net Change (%)
		Phase 1	Phase 2	Phase 3	Phase 1→3
orf1a	c70t(R24C)	0.05	5.35	0.23	+0.18
	t568c(F190L)	6.03	3.98	0.91	-5.12
	t794c(I265T)	11.29	7.40	4.19	-7.1
	g2748a(L916L)	0	0	5.18	+5.18
	c3508t(R1170C)	0	0.65	16.36	+16.36
	c4190t(A1397V)	0.03	0	5.18	+5.15
	c6821t(T2274I)	0.02	0.44	16.21	+16.19
	g7818a(M2606I)	0.03	2.16	27.09	+27.06
	c10054t(L3352F)	3.98	21.53	44.67	+40.69
	a10058g(K3353R)	0.19	6.10	8.60	+8.41
	c11651t(S3884L)	4.73	8.26	5.18	+0.45
	g11978a(R3993H)	0	0.02	5.10	+5.1
orf1b	t724c(L242L)	0.01	0.67	15.60	+15.59
	c1338t(Y446Y)	0.05	2.19	27.55	+27.5
	t1809a(P603P)	0.01	6.37	5.25	+5.24
	t2100c(S700S)	0	0	5.18	+5.18
	c2187t(D729D)	0.07	0.62	9.59	+9.52
	g2205t(E735D)	0.01	0.05	5.94	+5.93
	c2625t(Y875Y)	0.03	0.11	16.06	+16.03
	c2793t(C931C)	1.04	9.56	20.47	+19.43
	t2958g(L986L)	0.01	5.27	0.23	+0.22
	t3734c(L1245S)	0	0.02	5.02	+5.02
	g4254t(V1418V)	0.03	0.40	5.94	+5.91
	c4954t(P1652S)	0	0	5.02	+5.02
	a4957g(N1653D)	0.04	10.57	42.09	+42.05
	c5410t(L1804L)	6.78	4.38	0.99	-5.79
	g5518t(V1840F)	0.01	0.37	5.78	+5.77
	c7837t(R2613C)	0.06	10.02	40.64	+40.58
S	c1623t(F541F)	0.01	0.13	6.01	+6.00
	g2338c(E780Q)	0	0.41	15.98	+15.98
	a2691t(P897P)	0	0.64	16.06	+16.06
N	c162t(T54T)	0.01	0.06	5.18	+5.17
	c199t(P67S)	0.03	10.46	39.88	+39.85
	g454t(A152S)	0.01	0.08	6.32	+6.31
	c548a(S183Y)	1.03	9.53	20.70	+19.67
	g569t(S190I)	0.01	6.32	8.37	+8.36
	c596t(P199L)	0.04	10.26	39.80	+39.76
	g1129t(D377Y)	0.14	0.60	13.17	+13.03

Supplemental Table S5.

Non-associated Clade GR mutations that either persisted or emerged during 2020. Data were extracted where, during at least one Phase period, the frequency of a particular mutation was ≥5% compared to the clade Phase 1 majority consensus. The mutation specifies the nucleotide change as well as the amino acid change with coordinates of the gene and its product. Non-synonymous mutations are shown in red.

Gene	Mutation	Frequency (%) Phase 1	Phase 2	Phase 3	Net Change (%) Phase 1→3
orf1a	c48t(L16L)	12.50	5.41	4.17	-8.33
	a566g(N189S)	0	0.04	9.17	+9.17
	c671t(T224I)	0.12	1.13	5.83	+5.71
	c1023t(C341C)	0	0.12	8.75	+8.75
	t1297c(C433R)	0	0.54	15.42	+15.42
	g1770t(L590F)	0	1.67	19.17	+19.17
	g2994t(Q998H)	0.30	1.05	5.42	+5.12
	a2997g(T999T)	0	0.08	11.67	+11.67
	t3480c(H1160H)	0	0.08	9.17	+9.17
	t8907c(L2969L)	2.25	1.59	7.92	+5.67
	a10683g(R3561R)	2.25	1.59	7.92	+5.67
	c10712t(A3571V)	0	0.12	7.50	+7.50
	c10785t(V3595V)	0	0.23	8.75	+8.75
	c11409t(Y3803Y)	0.12	0.27	15.42	+15.30
	c11691t(D3897D)	0.06	1.94	18.75	+18.69
	c11760t(S3920S)	3.40	3.31	17.92	+14.52
	c13161t(R4387R)	0	0.23	9.58	+9.58
orf1b	c1470t(D490D)	0	0.23	8.75	+8.75
	c2466t(Y822Y)	14.32	26.60	4.58	-9.74
	g3346a(V1116I)	6.13	2.92	0	-6.13
	g3501t(E1167D)	11.95	24.27	4.58	-7.37
	c5544t(D1848D)	0.91	5.02	0	-0.91
	t6372c(N2124N)	22.82	38.12	32.92	+10.1
	c7007t(T2336I)	2.06	5.10	0	-2.06
S	g79t(A27S)	0	0.35	5.42	+5.42
	c249t(V83V)	0	0	8.33	+8.33
	g331a(D111N)	0	0.12	8.75	+8.75
	c1221t(V407V)	0	0.12	8.75	+8.75
	c2042a(P681H)	0	0.12	7.50	+7.50
	c2145t(P715P)	3.46	3.62	20.00	+16.54
	a2194g(T732A)	0.18	0.74	7.92	+7.74
	a3312g(V1104V)	0	0.12	7.50	+7.50
	c3342t(I1114I)	0.06	0.31	9.17	+9.11
M	g208t(V70F)	2.31	1.63	7.92	+5.61
	a405g(E135E)	0	0.12	7.08	+7.08
N	g105t(A35A)	0.55	1.32	8.75	+8.20
	c126a(P42P)	0	0.04	5.42	+5.42
	a783g(K261K)	0	0.23	5.83	+5.83

References

71. Le Bert, N.; Tan, A. T.; Kunasegaran, K.; Tham, C. Y. L.; Hafezi, M.; Chia, A.; Chng, M. H. Y.; Lin, M.; Tan, N.; Linster, M.; Chia, W. N.; Chen, M. I.; Wang, L. F.; Ooi, E. E.; Kalimuddin, S.; Tambyah, P. A.; Low, J. G.; Tan, Y. J.; Bertoletti, A., SARS-CoV-2-specific T cell immunity in cases of COVID-19 and SARS, and uninfected controls. *Nature* **2020**, 584, (7821), 457-462.
72. Nelde, A.; Bilich, T.; Heitmann, J. S.; Maringer, Y.; Salih, H. R.; Roerden, M.; Lübke, M.; Bauer, J.; Rieth, J.; Wacker, M.; Peter, A.; Hörber, S.; Traenkle, B.; Kaiser, P. D.; Rothbauer, U.; Becker, M.; Junker, D.; Krause, G.; Strengert, M.; Schneiderhan-Marra, N.; Templin, M. F.; Joos, T. O.; Kowalewski, D. J.; Stos-Zweifel, V.; Fehr, M.; Rabsteyn, A.; Mirakaj, V.; Karbach, J.; Jäger, E.; Graf, M.; Gruber, L. C.; Rachfalski, D.; Preuß, B.; Hagelstein, I.; Märklin, M.; Bakchoul, T.; Gouttefangeas, C.; Kohlbacher, O.; Klein, R.; Stevanović, S.; Rammensee, H. G.; Walz, J. S., SARS-CoV-2-derived peptides define heterologous and COVID-19-induced T cell recognition. *Nat Immunol* **2021**, 22, (1), 74-85.
73. Zheng, M.; Song, L., Novel antibody epitopes dominate the antigenicity of spike glycoprotein in SARS-CoV-2 compared to SARS-CoV. *Cellular & Molecular Immunology* **2020**, 17, (5), 536-538.
74. Shrock, E.; Fujimura, E.; Kula, T.; Timms, R. T.; Lee, I. H.; Leng, Y.; Robinson, M. L.; Sie, B. M.; Li, M. Z.; Chen, Y.; Logue, J.; Zuiani, A.; McCulloch, D.; Lelis, F. J. N.; Henson, S.; Monaco, D. R.; Travers, M.; Habibi, S.; Clarke, W. A.; Caturegli, P.; Laeyendecker, O.; Piechocka-Trocha, A.; Li, J. Z.; Khatri, A.; Chu, H. Y.; Villani, A. C.; Kays, K.; Goldberg, M. B.; Hacohen, N.; Filbin, M. R.; Yu, X. G.; Walker, B. D.; Wesemann, D. R.; Larman, H. B.; Lederer, J. A.; Elledge, S. J., Viral epitope profiling of COVID-19 patients reveals cross-reactivity and correlates of severity. *Science* **2020**, 370, (6520).
75. Oliveira, S. C.; de Magalhães, M. T. Q.; Homan, E. J., Immunoinformatic Analysis of SARS-CoV-2 Nucleocapsid Protein and Identification of COVID-19 Vaccine Targets. *Frontiers in Immunology* **2020**, 11, (2758).
